# Clinical and translational science and climate change: Time for action

**DOI:** 10.1017/cts.2022.9

**Published:** 2022-01-31

**Authors:** Julian Solway, Nicholas Kenyon, Lars Berglund

**Affiliations:** 1 Departments of Medicine and Pediatrics, University of Chicago, Chicago, IL, USA; 2 Department of Internal Medicine, University of California, Davis, Sacramento, CA, USA

The 10 plagues of Egypt [1] are reported in the Hebrew bible as a devastating public health consequence of harmful human behavior. While the secular historical record is uncertain about whether these horrible events actually happened three millennia ago, current consequences of human behavior resulting in climate change are most certainly real and bear uncanny similarity (with just a little poetic license) to those ancient plagues, as suggested by Döhler [2]:

*Blood* poisoned the waters of Egypt inducing widespread thirst, while climate change-induced droughts have likewise caused unslakable thirst. With the waters of the Nile poisoned, *frogs* had to leave their watery homes for more habitable conditions on land; climate change has made many regions less habitable, forcing people to abandon their homes in search of more livable places. *Insects* and *wild animals* plagued the ancient Egyptians, while global warming has increased the range of and human contact with mosquito and other disease vectors. The *pestilence* and *boils* of old are mirrored in expanded spread of existing and new infectious diseases among people and animals, while the *hail* that tormented the Egyptians has a modern parallel in the extreme weather events that have become commonplace. As *locusts* did back then, climate change now causes food insecurity through disruption of both land and marine food sources, and the *darkness* that ruined environmental quality then augurs the heat, smoke, and floods we suffer today. While we are not yet suffering widespread *death of the firstborns*, there has been a large increase in heat-related mortality among our elderly. Truly, the adverse health consequences of climate change are of biblical proportion, and while these climate events seem overwhelming, we emphasize the important role that clinical and translational research can play in bringing hope and better health for all as we face the future.

Our colleagues in other fields – the physical, environmental, and social sciences – have been grappling with this issue for some time; indeed, the 2021 Nobel prize in Physics was awarded for modeling of climate change. The Clinical and Translational Science community is also responding. More than 600 reviews on health and climate change have been published in the last 3 years, including >90 systematic reviews (summarized by the *British Medical Journal* [3]), a superb analysis from *The Lancet* [4], and a recent *JCTS* review by Stenvinkel et al [5] highlighting that loss of biodiversity may irreversibly rob us of valuable models and therapeutics from the animal and plant kingdoms.

Rising to the challenge, the National Academy of Medicine (NAM) has issued a call for action on climate change for a more sustainable, resilient future, and NAM President Victor Dzau pointed out the very real societal and public health threats posed by climate change in his 2020 presidential address. NAM is also partnering with the Burroughs Wellcome Fund in a Climate Change and Health Opportunity Grant initiative [6]. Project foci include safe drinking water, mental health effects on vulnerable populations, impact of climate change on children’s health and development, modeling impact of extreme events and temperatures on city dwellers, effects of increasing temperatures on nutritional quality and food availability, mental health impact of wildfires on residents and nontraditional firefighters, strengthening community health and developing strategies to reduce the carbon print of the health care sector. NIH/NCATS’s Clinical and Translational Science Award consortium is also addressing climate change; its nimble and effective response to the COVID-19 pandemic demonstrates the power that the CTSA consortium can bring to this problem. For example, attendees at its December, 2021 annual CTSA Program Meeting identified additional actions and studies the health sector can undertake, including raising educating and raising the awareness of health professionals and the public, engaging communities to mobilize to take action, developing predictive models of the effects of climate change on chronic diseases and health risk throughout the lifespan, developing Climate Change and Health as a viable field of study for young researchers, and many others. These efforts would best be undertaken as team science with colleagues in social, geospatial, and environmental sciences, behavioral economics, political science, and public policy. A so-called unmeeting on climate change has also been scheduled. We further recommend cataloging our present research inventory and coordinating future efforts to address health effects from repeated climate change events throughout our community of scientists; such efforts are likely to be impactful and could conceivably result in a multiplicative effect with increased synergy and visibility. *JCTS* would be delighted to consider such contributions, either self-standing or as part of a theme.

As Yogi Berra said, “it is getting late early.” Now is the time for clinical and translational scientists to take a lead in addressing these issues. The many challenges of health disparity and economic divide already cast long shadows over our ability to provide the care and public health resources necessary for everyone in the USA. The longer the climate change effects last, the more extensive the health problems will be and the more difficult it will be to ensure solutions. As scientists, we need to add our voice and participate prominently in the public discussion, engaging communities as well as in finding solutions. The community engagement program of clinical and translational science centers is of paramount importance and offers valuable discussion forums to engage with our fellow citizens.
